# Microbial pathogens associated with acute childhood diarrhoea in Kumasi, Ghana

**DOI:** 10.1186/s13104-017-2578-9

**Published:** 2017-07-11

**Authors:** Gilbert Kotei Ashie, Mohamed Mutocheluh, Michael Owusu, Theophilus Benjamine Kwofie, Samuel Akonor, Patrick Williams Narkwa, Samuel Blay Nguah, Joslin Dogbe

**Affiliations:** 1 Department of Clinical Microbiology, School of Medical Sciences, Kwame Nkrumah University of Science and Technology, Kumasi, Ghana; 2 Department of Clinical Microbiology, Komfo Anokye Teaching Hospital, Kumasi, Ghana; 3 Department of Child Health, Komfo Anokye Teaching Hospital, Kumasi, Ghana

**Keywords:** Childhood diarrhoea, Enteropathogens predominance, Kumasi-Ghana

## Abstract

**Background:**

Diarrhoeal diseases are among the most frequent causes of morbidity and mortality in children worldwide, especially in sub-Saharan Africa. This case–control study was conducted to investigate the bacterial, viral and parasitic pathogens associated with acute diarrhoea among children attending three health facilities in Kumasi, Ghana.

**Methods:**

Stool specimens were collected from 240 children under 5 years of age visiting hospitals in Kumasi, Ghana due to acute diarrhoea and from 107 healthy controls of similar age. Both intestinal and malaria parasites were diagnosed by microscopy whereas rota- and adenoviruses were identified by stool antigen immunochromatograhic testing. Bacterial enteropathogens were detected by conventional culture techniques.

**Results:**

Of all subjects, 23 (6.6%) were positive for malaria parasitaemia, 139 (40.1%) had at least one bacterial agent in their stool and 25 (7.2%) had ova or parasites. Subjects infected with malaria had the highest odds of having diarrhoea [12.0 (95% CI 1.56, 92.35)] followed by those with rotaviruses [4.4 (95% CI 2.05, 9.47)] and bacterial infection [4.99 (95% CI 1.45, 17.17)].

**Conclusion:**

In conclusion, this study was unique as it looked at the three groups of pathogens (parasites, viruses and bacteria) that cause acute diarrhoea in children in the Kumasi metropolis of Ghana. This study has shown for the first time since 2004 that malaria parasitaemia, rotavirus and bacterial infections still remain common pathogens associated with acute childhood diarrhoea in the Kumasi metropolis of Ghana.

## Background

Although diarrhoeal disease is highly preventable it accounts for approximately 9% of all deaths among children under 5 years of age globally [1]. This means about 580,000 child deaths due to preventable diarrhoea [2]. Recent reports suggest most deaths from diarrhoea occur among children less than 2 years of age living in South Asia and sub-Saharan Africa [3]. Reports from UNICEF indicate the total annual number of deaths from diarrhoea among children under five decreased by more than 50%—from over 1.2 million to half a million in the last 15 years (http://data.unicef.org/topic/child-health/diarrhoeal-disease/#). Although the mortality rate associated with diarrhoea is decreasing worldwide, cases in sub-Saharan Africa continue to rise because of factors such as unsanitary handling of food, malnutrition, inadequate safe drinking water and general lack of personal hygiene [3].

It is known that the combined effect of malnutrition and continuous diarrhoeal episodes culminate in impairment of cognitive development and work rate of children [4].

Childhood diarrhea is globally reported to be associated with a wide range of bacteria including *Escherichia coli*, *Salmonella* spp., *Vibrio cholera;* enteroparasites including *Entamoeba histolytica, Giardia lamblia, Cryptosporidium parvum* and viruses including adenoviruses and rotaviruses [5, 6]. However, much attention seems to focus on rotaviruses without recognition for other enteroparasites, haemoparasites and bacteria [7, 8].

There is paucity of data on the aetiological agents of childhood diarrhoea in the Kumasi metropolis in Ghana in the past 10 years. The study was therefore conducted to describe the pathogens associated with acute childhood diarrhea in three health facilities in Kumasi, Ghana.

## Methods

### Study area

The study was conducted at three health facilities in the Kumasi metropolitan area. The facilities were Kumasi Maternal and Child Health Hospital, Kwame Nkrumah University of Science and Technology (KNUST) Hospital and the Manhyia Government Hospital. The Kumasi metropolitan area has nine sub-municipalities. It is a cosmopolitan urban city of the Ashanti Region of Ghana, located in the transitional forest zone and is about 270 km north of the national capital Accra. The region has a geological altitude between 250 and 300 feet above sea level and a population of about 1,889,934 (www.ghanadistricts.com/districts).

The Maternal and Child Health Hospital, KNUST Hospital and the Manhyia Government Hospital in Kumasi are known to record high visit rates for children under the age of 5 years.

### Study design and sample size

This was a case–control study carried out between September 2012 and April 2014 among children 5 years and below presenting to three health facilities in Ghana.

Case groups were defined as subjects with three or more watery or loose stools within 24-h period prior to admission to the hospital. Controls were subjects attending clinic for reasons other than diarrhoea and presenting with no history of diarrhoea for at least 21 days prior to visiting the health facility. Prior to the study, a sample size of at least 208 cases and 104 control groups was determined taking into consideration a two sided alpha level of 5%, study power of 80%, assumed proportion of 60% pathogens detection among cases and 42.5% among controls. The proportions of cases and controls were extrapolated based on hospital records of diarrhea cases. This could reduce the power to detect differences for specific pathogens unless this difference is large. Sample size calculation for cases and control was based on Fisher exact test.

All children presenting to the facilities were assessed by general practitioners and those who qualified as cases were selected (about 10 per week on average) until the required number was reached. After every second case that was recruited, a control was selected.

### Study site, sample collection and storage

For all subjects, stool and blood samples were collected after informed consent was sought from their parents/guardians evidenced by the completion and signing or thumb-printing the informed consent forms. The socio-demographic characteristics were also entered on standard questionnaires by research assistants and the texture of the stool samples were recorded in the laboratory books.

Stool samples were collected using plastic applicators attached to the lid of the stool containers into clean disinfectant free containers. The containers were closed tightly, labelled with patient’s details and shipped immediately to the research laboratory of the Department of Clinical Microbiology of the School of Medical Sciences, Kwame Nkrumah University of Science and Technology. Samples that were not processed immediately were stored in the refrigerator or frozen. Subjects who could not pass stools or had any form of therapeutic intervention within 48 h before reporting to the hospital were excluded. Therefore, the 347 subjects included in the study was the number left after exclusions for not passing stools and having a therapeutic intervention within 48 h.

### Stool culture and viral antigen detection

Samples were microscopically examined for parasites using physiological saline (0.85%) wet preparation. Stool specimens were cultured using standard conventional culturing techniques within 2 h of specimen collection. For the culturing methods, a loop full of the stool was directly inoculated onto MacConkey agar and about 3 g of the same was inoculated into Selenite faecal broth following standard operating procedure for broth inoculation. The plates and broths were incubated aerobically at 37 °C for overnight and for 18 h respectively. Sub-culture from Selenite faecal broth was made onto Salmonella–Shigella agar and incubated aerobically at 37 °C overnight after which the biochemical tests including; Urea, Triple sugar iron agar tests, Indole, and Citrate tests were done to identify the organism(s) where necessary.

The presence of viral pathogens were examined using “*Diagnostic Automation Cortez*” *Adeno/Rota combo qualitative* rapid antigen card test (USA) which is based on the sandwiched solid phase immumochromatographic assay technique and following the manufacturer’s instructions. Briefly, samples and test kits were allowed to thaw and equilibrated to room temperature. The bottles containing the samples were unscrewed and the attached applicator sticks used to transfer small piece of stool (50–200 mg) or 100 µl (for liquid or semi-solid stool) to the vial containing specimen preparation buffer after which the caps were replaced. The buffer-stool mixtures were mixed thoroughly by shaking the bottles for a few seconds. The test cards were then removed from the sealed foil pouch and while holding the samples upright with the tip point towards the direction away from the test performer, the tips were snapped off. The bottles were held in a vertical position over the sample well of the test card, and three drops (120–150 µl) of the diluted sample were delivered into the sample well and the results read between 5 and 10 min according to the manufacturer’s instructions.

The Adeno/Rota combo test kit used in this study is limited because: (i) rapid test kits are generally associated with false results i.e. the results of these rapid test kits require confirmation with superior techniques such as RT-PCR or virus culture, (ii) the control materials to validate the reliability of the device were not provided by the test kit and (iii) cannot differentiate between the virus strains.

### Detection of malaria parasites

The gold standard for diagnosing malaria parasite infection is still based on finding *Plasmodium* trophozoites in blood films. Blood samples from heel or finger prick from the middle finger were taken directly onto clean glass slides and film prepared by using an applicator to form a circular pool thick enough to allow print letters to be read through and allowed to air-dry. The dried slides were flooded with freshly made Giemsa stain diluted 1 in 10 with buffered distilled water (pH 7.2). The slides were allowed to stain for 15–30 min. Tap water was scuttled over the slides to wash off the stains after which the slides were drained and dried vertically. The film was examined according to a standard procedure.

### Ethical considerations and consent to publish

The protocol of this study was approved by the Committee on Human Research Publication and Ethics of Kwame Nkrumah University of Science and Technology. Informed consent was obtained from all parents or guardians prior to enrolment. Parents who refused to consent were excluded from the study.

### Statistical analysis

All data obtained from this study were entered using Microsoft Excel spreadsheet. Data was imported and analysed using R statistical software version 3.2.1 [9]. For the purposes of analysis, all stool bacterial commensals were coded as “no bacterial growth” while pathogens including *Salmonella* species and *Shigella* species were coded as bacterial pathogens. The commensal bacteria recoded as “no bacterial growth” were *Klebsiella ozanea, Klebsiella pneumonea, Proteus* spp., *Klebsiella aerogenes*, *Pseudomonas aeruginosa, Proteus mirabilis* and *Proteus vulgaris*. *E. coli* was also considered as commensal because of our inability to distinguish the pathogenic from the non-pathogenic ones through serotyping.

Stool samples positive for hookworm species, *Giardia* spp., *Strongyloides* spp. and *Taenia* species were also recoded as “stool positives”.

All categorical possible risk factors and their association with diarrhoea were first analysed using a univariable logistic regression with the risk factors as the predictors and presence of diarrhoea as the outcome. The possible risk factors that were significantly associated with diarrhoea defined as p < 0.3 were entered into a multivariable logistic regression model using a forward stepwise approach for selection of significant variables while adjusting for all other variables in the model. An interaction term for the risk factors was also included in the model where appropriate in order to assess for possible interaction. Only age and the variables listed in Table 2 were used as covariates in the regression modelling. All associations between the risk factors and diarrhoea were expressed as the adjusted odd ratios (OR) and 95% confidence interval (CI) and plotted using sjPlot package in R [10].

## Results

### Demographics

Three hundred and forty-seven subjects (347) made up of 240 cases and 107 controls were enrolled in this study after exclusions for not satisfying the inclusion criteria for cases and controls. Of the 240 cases, 152 (63.3%) were males and 88 (36.7%) were females. The median age of cases was 14.0 (IQR = 8.5–24.0). Of the 107 controls, 65 (60.7%) were males and 42 (39.3%) were females. The median age of controls was 13.0 (IQR = 7.5–24.0).

### Distribution of pathogens

Twenty-three subjects (6.6%) were positive for malaria parasitaemia, 139 (40.1%) had at least one bacterial agent and 25 (7.2%) had ova or parasites in their stool samples. Seventy-nine (73.8%) controls and 112 (46.7%) cases had no pathogen identified in their samples. Twenty-five (23.3%) controls and 42.5% cases had exactly one pathogen identified, 3 (3.8%) controls and 24 (10%) cases had exactly two pathogens and two cases had more than three pathogens identified. Table 2 describes pathogens identified stratified by cases and controls.

Most of the adenoviruses were identified in the 1st year of life whereas rotaviruses occurred predominantly in children between the ages of 13 and 24 months. Stool parasites occurred more frequently in older children compared to younger ones whereas malaria increased steadily from younger age groups to older ones. A Chi squared test for trend in proportions among acute diarrhoea children showed a significant association between increasing age categories and increasing percentage positivity of stool parasite (p value = 0.0003) and malaria (p value = 0.009) exposures whereas decreasing adenoviruses positivity were associated with increasing age (p value = 0.013). Table 1 describes the pathogens identified in each age category.

**Table 1:** Distribution of age categories and pathogens isolated

Age categories	0–12	13–24	25–60	Total	p values
Total	155	132	60	347	
Status of malaria by microscopy					0.02
Positive, n (%)	4 (2.6)	12 (9.1)	7 (11.7)	23 (6.6)	
Organisms isolated by culture					<0.001
*Salmonella* spp., n (%)	7 (4.5)	2 (1.5)	11 (18.3)	20 (5.8)	
*Shigella* spp., n (%)	5 (3.2)	8 (6.1)	3 (5)	16 (4.6)	
Adenoviruses identified in stool specimen					0.005
Positive, n (%)	23 (14.8)	6 (4.5)	3 (5)	32 (9.2)	
Rotaviruses identified in stool specimen					<0.001
Positive, n (%)	16 (10.3)	42 (31.8)	14 (23.3)	72 (20.7)	
Binary variable for stool positivity					<0.001
Positive, n (%)	4 (2.6)	9 (6.8)	12 (20)	25 (7.2)	
Whether bacterial grew in sample					0.001
Positive, n (%)	12 (7.7)	10 (7.6)	14 (23.3)	36 (10.4)	

A cross tabulation of stool appearance with enteropathogens showed that watery diarrhoea occurred in 4 *Salmonella* spp. positive subjects, 2 *Shigella* spp. positive subjects, 1 *Ancylostoma* species positive subject and 2 *Hymenolepsis* species positive subjects. There was no statistically significant association between type of enteropathogen and appearance of stool samples. Compared to control subjects, malaria (p = 0.009), rotaviruses (p < 0.001) and presence of bacteria (p < 0.001) were significantly associated with diarrhoea. Table 2 describes the distribution of enteric pathogens in case and control subjects.

**Table 2:** Pathogens associated with acute diarrhoea

Pathogens *n (%)*	Controls (n = 107)	Cases (n = 240)	Odds ratio	Total (n = 347)
*Parasites*				
Blood sample				
Malaria parasite positive	1 (0.9)	22 (9.2)	10.7	23 (6.6)
Stool samples				
Microscopy			0.54	
*Ancylostoma* species	0 (0)	1 (0.4)		1 (0.3)
*Giardia* species	5 (4.7)	7 (2.9)		12 (3.5)
*Hymenolepsis* species	2 (1.9)	2 (0.8)		4 (1.2)
*Strongyloides* species	4 (3.7)	4 (1.7)		8 (2.3)
*Bacteria*			5.53	
Cultures				
*Salmonella* spp.	2 (1.9)	18 (7.5)		20 (5.8)
*Shigella* spp.	1 (0.9)	15 (6.2)		16 (4.6)
Viruses				
Rapid test				
Adenovirus positive	7 (6.5)	25 (10.4)	1.66	32 (9.2)
Rotavirus positive	9 (8.4)	63 (26.2)	3.88	72 (20.7)

### Risk factors associated with acute diarrhoea

**Fig. 1:**
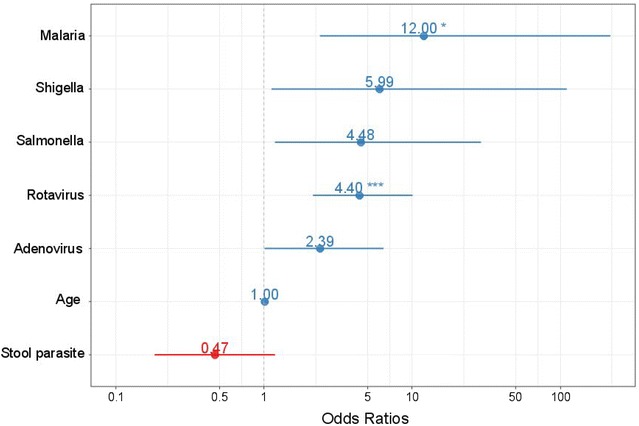
Pathogens associated with acute diarrhoea. The *blue dots* represent odds ratios of pathogens greater than 1 and *red dots* represent odds ratios less than 1. The *bars* represent a two sided 95% confidence intervals. Level of statistical significance are shown by ***p < 0.001 and *0.02

The variables that were included as covariates in the regression modelling after preliminary analysis of the data were malaria infection, rotavirus infection, *Salmonella*, *Shigella*, adenovirus infection, stool parasitic infection and age. A logistic regression analysis using the diarrhoea status as outcome and type of pathogen as predictor showed that subjects infected with malaria had the highest odds of having diarrhoea [adj. OR = 12.0 (95% CI 1.56, 92.35)] followed by *Shigella* infection [adj. OR = 5.99 (95% CI 0.75–47.64)], *Salmonella* infection [adj OR = 4.48 (95% CI 0.98–20.47)] and rotaviruses [adj OR = 4.4 (95% CI 2.05–9.47)]. There was no significant interaction among any of the risk factors. Figure 1 describes pathogens associated with acute diarrhoea.

## Discussion

Acute diarrhoea is one of the most common diseases and causes of death in young children worldwide [1]. The current study focused on the microbial pathogens associated with acute diarrhoea among children aged 5 years and below who visited some major hospitals within the Kumasi metropolis of Ghana. The current study suggested rota- and adenoviruses were the commonest diarrhoea causing pathogens emphasizing the important role of these pathogens in causing childhood diarrhoea and consistent with studies from Ghana and beyond [5, 11, 12]. Compared to control subjects, it was observed that being positive for any bacteria (*Salmonella* spp. or *Shigella* spp.) were significantly associated with diarrhoea. Similar studies in Asia and sub-Saharan Africa have identified these pathogens mostly in diarrhoea patients [13–16]. A study conducted by Rauthaur et al. in India and aimed at determining the prevalence of various bacterial enteropathogens causing acute childhood diarrhoea showed *Salmonella* and *Shigella* were major contributors to the severe diarrhoea accounting for 28.2% and 13.2% respectively [17].

Notable among the stool parasites identified were *Gardia* species, *Ancylostoma* spp. and *Strongyloides* spp. There was however no significant difference in parasite detections between case and control groups. Interestingly, this study shows there were more *Giardia* spp. in the controls than the cases consistent with findings from Tellevik et al. [18], who reported a *Giardia lamblia* prevalence case to control ratio of 3.4–6.1% in a study that investigated the prevalence of *C. parvum/hominis, E. histolytica* and *G. lamblia* among young children in Dar es Salaam, Tanzania. The role of *G. lamblia* in causing diarrhoea symptoms in endemic areas is controversial and studies are now reporting that *G. lamblia* may not cause diarrhoea in endemic areas like Africa [18]. More so, the association between stool parasites and occurrence of diarrhoea has continued to attract mixed reviews. Whereas some authors found no relationship between diarrhoea and presence of stool parasite [18, 19], other studies reported otherwise [20]. Further studies to explore the role of enteroparasites in diarrhoea subjects would be of immense benefit to public health.

Our study also identified significant association between exposure to malaria and acute childhood diarrhoea. This phenomenon has been reported in other subjects [21, 22] and emphasizes the important role of malaria parasitemia in causing diarrhoea among children. It is important to note that malaria still account for significant childhood diarrhoea although other enteropathogens also have a role.

The few limitations in this study include our inability to type the bacterial strains of *Salmonella enterica* spp. and *Shigella* spp. due to logistic constraints. Also, the results of the virus detection kit should have been confirmed using some superior techniques such as RT-PCR or virus culture because rapid test kits are generally linked with false positive detection of microbial antigens. It should also be noted that the power of our study was calculated based on the prevalence of any pathogens and that the study had low power to find significant differences between prevalences of specific pathogens in cases and controls unless the difference was large. Although it would have been helpful to include data on rotavirus vaccination, this was not done because of the limited records on the history of vaccination.

It is therefore recommended that future studies to target larger populations with the use of advanced techniques such as RT-PCR to detect subtypes of viruses and bacteria in stool samples. A study on the prevalence of rotavirus among vaccinees should be conducted in the future to ascertain the success or otherwise of the immunization programme.

## Conclusion

This study was unique as it looked at the three groups of pathogens (parasites, viruses and bacteria) that cause acute diarrhoea in children in the Kumasi metropolis of Ghana. This study has shown for the first time since 2004 that malaria parasitaemia, rotavirus and bacterial infections still remain common pathogens associated with acute childhood diarrhoea in the Kumasi metropolis of Ghana.

## Abbreviations

KNUST: Kwame Nkrumah University of Science and Technology
IQR: interquartile range
CI: confidence interval
OR: odds ratio
